# The Denitrification Characteristics of *Pseudomonas stutzeri* SC221-M and Its Application to Water Quality Control in Grass Carp Aquaculture

**DOI:** 10.1371/journal.pone.0114886

**Published:** 2014-12-09

**Authors:** Bin Deng, Luoqin Fu, Xiaoping Zhang, Jiajia Zheng, Lisha Peng, Jiandong Sun, Haiyan Zhu, Yibing Wang, Weifen Li, Xuexiang Wu, Di Wu

**Affiliations:** 1 Key Laboratory of Molecular Feed Science, Institute of Animal Nutrition and Feed Science, College of Animal Science, Zhejiang University, Hangzhou, China; 2 School of Pharmaceutical Engineering, Guizhou Institute of Technology, Guiyang, China; 3 College of Biosystems Engineering and Food Science, Zhejiang University, Hangzhou, China; 4 College of Animal Science, Guizhou University, Guiyang, China; 5 Center for Disease Control and Prevention, Deyang, China; Oak Ridge National Laboratory, United States of America

## Abstract

To reduce ammonium and nitrite in aquaculture water, an isolate of the denitrifying bacterium *Pseudomonas stutzeri,* SC221-M, was obtained. The effects of various nitrogen and carbon sources, the ratio of carbon to nitrogen and temperature on bacterial growth, denitrification rates and the expression levels of *nirS* and *nosZ* in SC221-M were studied. The following conditions were determined to be optimal for growth and denitrification in SC221-M: NaNO_2_ as the nitrogen source, sodium citrate as the carbon source, a carbon to nitrogen ratio range of 4–8, and a temperature range of 20–35°C. Subsequently, SC221-M and the *Bacillus cereus* BSC24 strain were selected to generate microbial preparations. The results showed that addition of the microbial preparations decreased various hydrochemical parameters, including total dissolved solids, ammonium, nitrite, total nitrogen and the chemical oxygen demand. Nitrogen removal rates were highest on day 9; the removal rates of BSC24, SC221-M, a mixed preparation and a 3× mixed preparation were 24.5%, 26.6%, 53.9% and 53.4%, respectively. The mixed preparation (SC221-M+BSC24) was more effective at removing nitrogen than either the SC221-M or BSC24 preparation. Roche 454 pyrosequencing and subsequent analysis indicated that the control and other groups formed separate clusters, and the microbial community structure in the water changed significantly after the addition of microbial preparations. These results indicate that the addition of microbial preparations can improve both the water quality and microbial community structure in an experimental aquaculture system. *P. stutzeri* strain SC221-M and its related microbial preparations are potential candidates for the regulation of water quality in commercial aquaculture systems.

## Introduction

An artificial pond is a simple ecosystem. When the pollutant load in an aquaculture system exceeds its capacity for self-purification, ammonium, nitrite and other pollutants accumulate, damaging the health of aquatic animals [1]. In particular, a high concentration of ammonium can damage the gill epithelial cells and weaken the immune systems of fish [2]. A high concentration of nitrite can be poisonous because it increases the level of methemoglobin in the blood, resulting in tissue hypoxia, nerve palsy, or even suffocation and death [3]. Water exchange, dredging, and the addition of lime and algaecides are common strategies applied to remove nitrogen and other pollutants. However, these approaches are expensive, may cause secondary pollution, and show additional disadvantages.

The addition of microorganisms to aquaculture systems has recently become a widespread practice for reducing nitrogen pollution and improving water quality. Nitrogen cycling in aquaculture systems is mainly accomplished by ammonifying bacteria, nitrifying bacteria, and denitrifying bacteria. Many microorganisms, including photosynthetic bacteria [4], nitrifying bacteria [5], denitrifying bacteria [6], and some species of Bacillus [7], have been used for commercial denitrification.

*P. stutzeri*, which belongs to the genus *Pseudomonas*, is widely found in soil, fresh water, oceans and animals. It is an aerobic Gram-negative bacterium and a type of denitrifying bacterium [8].

A variety of strains of *P. stutzeri* have been isolated to study environmental bioremediation. *P. stutzeri* is capable of degrading a number of organic pollutants, such as naphthalene [9], chloronaphthalene, methylnaphthalene, chloro-salicylate, and methyl salicylate [10]. *P. stutzeri* can also convert hexavalent chromium, precipitated mercury, cadmium, lead, arsenic and other elements [11]. Additionally, *P. stutzeri* is capable of nitrogen fixation [12]. There are a number of advantages to using *P. stutzeri* for environmental remediation and recovery.

Previous studies have shown that *P. stutzeri* can perform denitrification in the presence of high oxygen levels [13] and can carry out nitrification and denitrification simultaneously. Genome analysis of *P. stutzeri* [12] has led to the identification of numerous genes involved in this process, including ammonia mono-oxygenase (*amo*), nitrite reductase (*nir*), nitrous oxide reductase (*nos*), and nitrate reductase (*nar*). However, little is known about the suitability of *P. stutzeri* for the remediation of aquaculture water.

In the present study, we investigated the denitrification and genomic characteristics of *P. stutzeri* SC221-M and the effects of a SC221-M microbial preparation on the water quality and the microbial community structure in an experimental aquaculture system to provide an experimental basis for using it to reduce nitrogen pollution.

## Materials and Methods

### 2.1 Isolation of bacteria

To reduce ammonium and nitrite in aquaculture water for grass carp, the denitrifying bacteria *P. stutzeri* SC221 (S1A Figure) and *B. cereus* BSC24 (S1B Figure) were isolated from a grass carp pond in Shaoxing, Zhejiang Province, China, according to the method described by Zhang *et al*. (2013) [7]. The bacteria were identified using Bergey’s Manual of Systematic Bacteriology and 16S rRNA gene sequences [14].

After cobalt-60 radiation and screening, the nitrite removal capacity of SC221 was increased from 50 mg/L to 100 mg/L. The strain obtained following radiation treatment was designated *P. stutzeri* SC221-M. The two strains were stored at −80°C using 20% glycerol as a cryoprotectant at the Institute of Animal Nutrition and Feed Science, Key Laboratory of Molecular Feed Science, Zhejiang University.

### 2.2 Nitrogen removal and quantitative real-time PCR

SC221-M was grown on LB plates for recovery. A purified colony was inoculated into 5 mL of broth, followed by incubation with reciprocal shaking at 30°C for 24 h. The cells were then centrifuged (4,000×*g*, 10 min), and the pellet was washed 3 times with a 0.85% saline solution. The washed pellet was resuspended in the 0.85% saline solution and mixed thoroughly. Subsequently, 10% SC221-M (resuspended in 0.85% saline) was used to inoculate denitrification media (S1 Table). Three replicates were employed for each treatment, and non-inoculated samples were used as controls. The samples were incubated at 30°C and shaken at 200 rpm for 24 h, then centrifuged at 8,000×*g* for 5 min. The supernatants were subsequently used to measure the levels of ammonium, nitrite, nitrate, and total inorganic nitrogen (TIN), according to standard methods [7], and the pellets were kept at −80°C before RNA extraction and the determination of *nirS* and *nosZ* genes.

An RNA extraction kit and reverse transcription kit were obtained from Haogene Ltd. (Hangzhou, Zhejiang, China). Total RNA extraction and reverse transcription were performed according to the manufacturer’s instructions. Using a LightCycler (Roche, Mannheim, Germany, quantitative PCR was performed to determine the expression levels of the *nirS* and *nosZ* genes of SC221-M using the set of primers shown in S2 Table. Each PCR tube contained 10.5 µL of ddH_2_O, 12.5 µL of 2× SYBR Premix ex Taq^TM^ (Sangon, Shanghai, China), 0.5 µL of the forward primer (10 µM), 0.5 µL of the reverse primer (10 µM), and 1 µL of template DNA. PCR amplification was performed with an initial 95°C denaturation for 1 min, followed by 45 cycles of 10 sec at 95°C and 25 sec at 62°C. Expression levels were computed relative to the 16S rRNA gene and expressed as 2^ΔCt^×10^4^.

### 2.3 Microbial preparations and water quality control

*P. stutzeri* SC221-M and *B. cereus* BSC24 were grown on LB plates for recovery. LB broth was inoculated with the purified strains and incubated in a shaker (200 rpm) at 30°C for 24 h for fermentation. The cells were harvested via centrifugation (4,000×*g*, 10 min, 10°C) and washed three times with a sterile 0.85% saline solution. The pellets were dried at 50–60°C to a dry powder. These preparations were stored at room temperature, and their purity was determined through the plate spreading technique. The final density was approximately 3×10^9 ^CFU/g of dry powder.

Fifteen circular polyethylene buckets (2,000 L) were filled with 1,400 L of fresh water from the Dafan River (Shaoxing, Zhejiang, China, 30° 03′ 38″ N, 120° 40′ 52″ E), and 1 kg of healthy grass carp (initial body weight: 15.55±1.04 g each) was transferred to each bucket. The fish were acclimatized for 7 days and then divided into five groups. The control group did not received any microbial preparation. The other four groups were given daily supplements (for 6 days) of 1) *B. cereus* BSC24 at a concentration of 1×10^5 ^CFU/mL; 2) *P. stutzeri* SC221-M at 1×10^5 ^CFU/mL; 3) a mixed preparation (SC221-M:BSC24 = 1∶1) at 1×10^5 ^CFU/mL; or 4) a 3× concentration of the mixed preparation at 3×10^5 ^CFU/mL in the water. The fish from each group were fed a basal diet (S3 Table) twice per day, at 9∶00 and 17∶00. The daily amount of feed provided was approximately 4–5% of the body weight of the fish. The trial lasted for 9 days. Water samples were collected every 3 days for the determination of total dissolved solids (TDS), ammonium, nitrite, nitrate, total nitrogen and chemical oxygen demand (COD).

### 2.4 Ethics statement

No specific permissions were required for the field studies. No specific permits were required for the Dafan River in Shaoxing, Zhejiang Province, China; this river is not privately owned or protected in any way. The field studies did not involve endangered or protected species. This study has been approved by the Animal Ethics Committee of the Institute of Animal Nutrition and Feed Science, College of Animal Science, Zhejiang University.

### 2.5 454 pyrosequencing and analysis

Twelve water samples were collected from the control and the other treatment groups, filtered through 0.22-µm filters (Sartorius, Germany), placed in sterile 2-mL collection tubes, and stored at −80°C before DNA extraction. Genomic DNA was extracted with a Genomic DNA Isolation Kit (Sangon, Shanghai, China). The V3 region of the 16S rRNA gene was amplified using the primers listed in S4 Table. We designed 12 barcode sequences of 7 nucleotides at the 5′ end of the forward primers to identify each sample. Each PCR tube contained 8.8 µL of ddH_2_O, 10 µL of 2× SYBR Premix ex Taq^TM^ (Sangon, Shanghai, China), 0.4 µL of the forward primer (10 µM), 0.4 µL of the reverse primer (10 µM), and 0.4 µL of template DNA. PCR amplification was performed with an initial 94°C denaturation for 5 min, followed by 35 cycles of 94°C for 40 sec, 52°C for 50 sec and 72°C for 40 sec. The resultant DNA fragments were separated via electrophoresis on 1% agarose gels and excised for purification using a gel extraction kit (Axygen Scientific Inc., USA). The DNA concentration was measured with a UV-vis spectrophotometer (NanoDrop ND1000, USA) and then adjusted to 50 ng/µL for each sample. Finally, equal amounts of DNA from each sample were mixed together and sequenced at Tongji-SCBIT Biotechnology Co. Ltd. Shanghai, China, using the 454 Life Sciences/Roche GS-FLX sequencing system (Roche Applied Science, Penzburg, Germany).

Sequencing reads and quality scores were obtained through 454 pyrosequencing. For sequence analysis, Qiime software was employed [15]. DNA sequence reads were filtered using the default parameters. In this step, low-quality or ambiguous reads were discarded, and primer sequences and barcodes were trimmed from the 5′ region. Operational taxonomic units (OTUs) were selected at the level of 97% identity, and a representative sequence from each OTU was classified directly with the Ribosomal Database Project (RDP) Classifier (version 2.2) [16]. Diversity between the samples (beta diversity) was assessed using the unweighted Unifrac test [17] and the unweighted pair-group method with arithmetic means (UPGMA). Finally, the microbial community structure in each sample at the phylum level was determined using Megan (version 4.70.4) [18].

### 2.6 Statistical analysis

Statistical analysis was performed with Microsoft Excel and SPSS version 16.0. All results are shown as the mean ± standard deviation. Statistical analyses of the data were conducted via one-way analysis of variance (ANOVA). *P*<0.05 was considered statistically significant (lowercase letters indicate significant differences). Differences showing *P<*0.01 were considered to be highly significant (uppercase letters indicate highly significant differences).

## Results and Discussion

### 3.1 Potential of *P. stutzeri* SC221-M to perform simultaneous heterotrophic nitrification and aerobic denitrification

**Figure 1:**
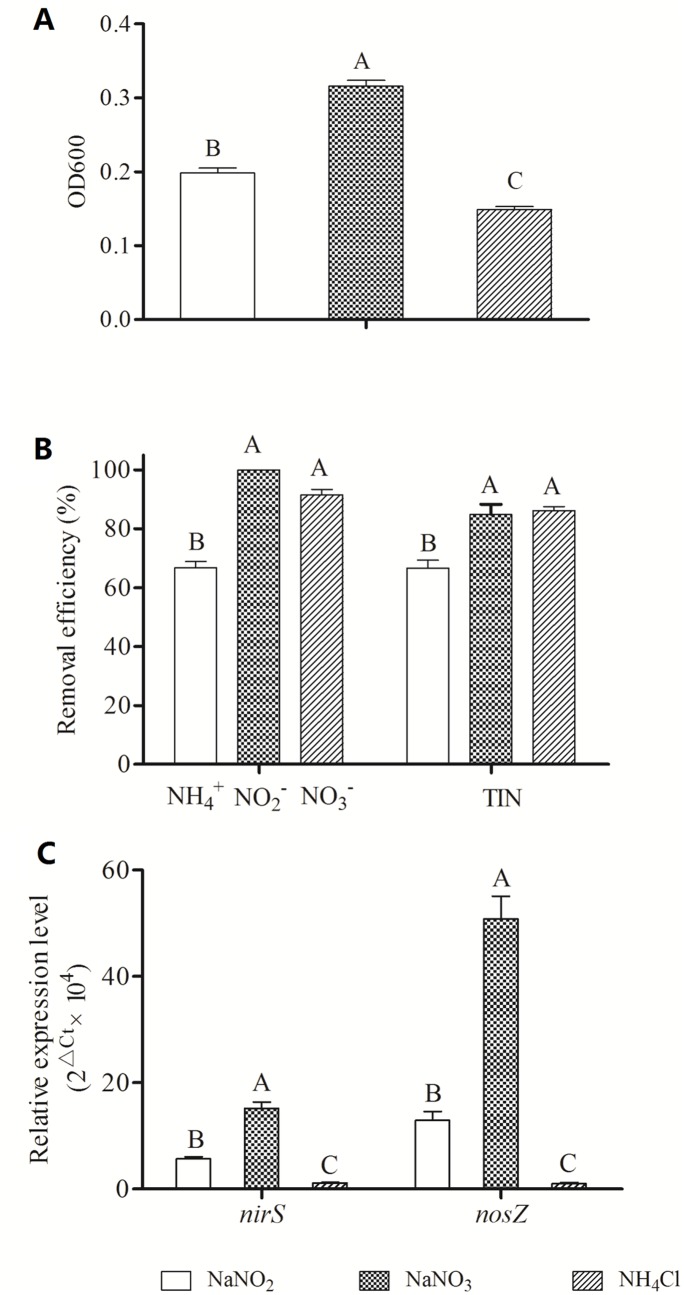
Effects of different nitrogen sources on the growth (A), denitrification (B) and gene expression (C) of SC221-M. Uppercase letters indicate highly significant differences (*P<*0.01); and lowercase letters indicate moderately significant differences (*P*<0.05). The same definitions apply to Fig. 3, Fig. 4 and Fig. 5.

When NH_4_Cl, NaNO_2_, or NaNO_3_ was used as the sole nitrogen source, SC221-M removed 66.79% of the ammonium, 99.88% of the nitrite, and 91.46% of the nitrate, respectively, which corresponds to total nitrogen removal efficiencies of 66.58%, 84.65%, and 86.12% (Fig. 1B). The removal rate of ammonium was significantly lower than that of nitrate or nitrite (*P<*0.01).

The quantitative real-time PCR results (Fig. 1C) showed that the presence of NaNO_2_ increased *nirS* and *nosZ* expression to a greater extent than the other two nitrogen sources (*P<*0.01). In contrast, previous studies in *Pseudomonas mandelii* indicated that nitrate promoted *nirS* expression but had little effect on *nosZ* expression [19].

Recent studies have identified bacterial strains that are capable of simultaneous heterotrophic nitrification and aerobic denitrification (using ammonium as a nitrogen source). The numerous species capable of heterotrophic nitrification and aerobic denitrification include *B. licheniformis, B. cereus* [20], *B. methylotrophicus* [21] and *P. stutzeri* [22]. These bacteria transform ammonium directly into nitrogen without accumulating nitrogen oxide intermediates. Our results suggest that *P. stutzeri* SC221-M is also capable of simultaneous heterotrophic nitrification and aerobic denitrification.

### 3.2 Effects of carbon sources on growth, denitrification and gene expression

**Figure 2:**
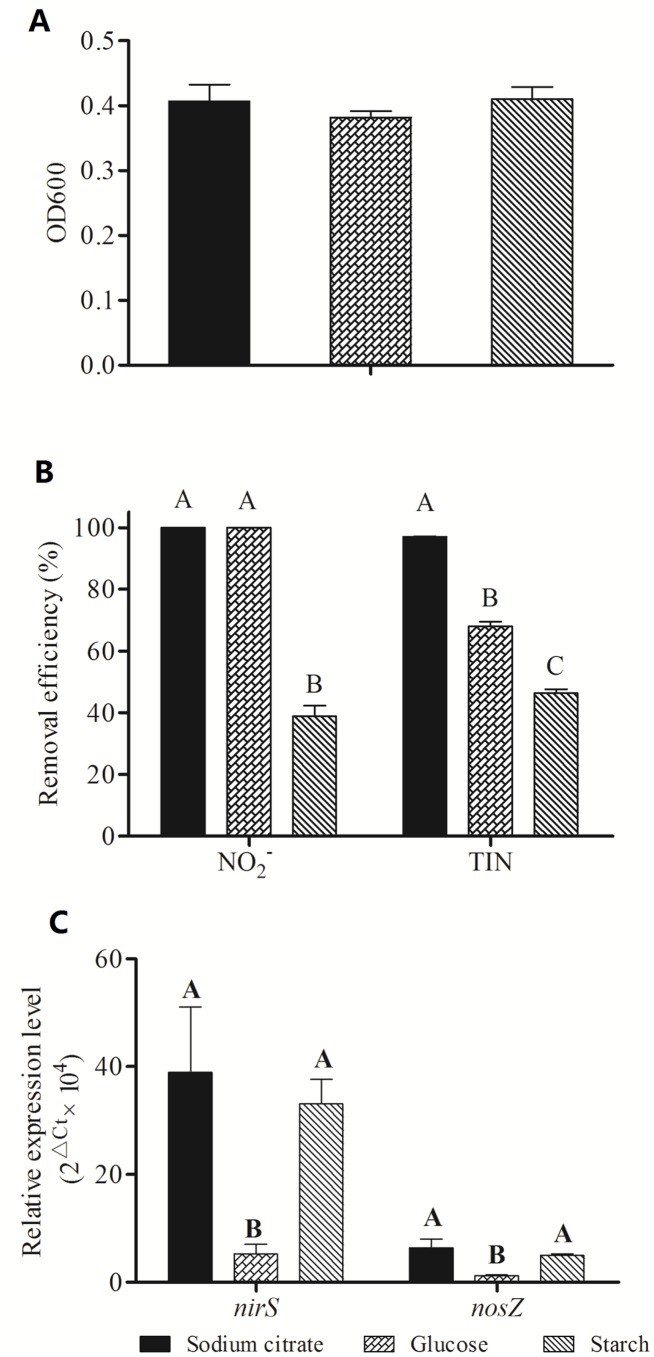
Effects of different carbon sources on growth (A), denitrification (B) and gene expression (C) in SC221-M.

As shown in Fig. 2A, SC221-M grew well in media containing sodium citrate, glucose or starch. The efficiency of total inorganic nitrogen removal associated with each carbon source was ranked as follows: sodium citrate> glucose> starch (Fig. 2B). When sodium citrate or starch was used as the carbon source, the expression levels of *nirS* and *nosZ* were significantly (*P<*0.01) higher than in the glucose group (Fig. 2C).

In general, denitrification can be promoted by using more available carbon sources. Hu *et al.* (2013) reported that the production rates of N_2_O were 2.8%, 5.5% and 8.8% when sodium acetate, glucose or soluble starch was used as the carbon source, respectively [23]. These results are consistent with our findings.

In the present study, glucose enhanced the expression of *nirS* and *nosZ,* but not as efficiently as sodium citrate and starch. These findings are similar to those of Henderson *et al.* (2010), who discovered that the denitrification rate and respiration increased after 48 h of incubation in glucose, whereas *nirS* and *nosZ* mRNA levels did not change [24].

### 3.3 Effects of the carbon to nitrogen (C/N) ratio on growth, denitrification and gene expression

**Figure 3:**
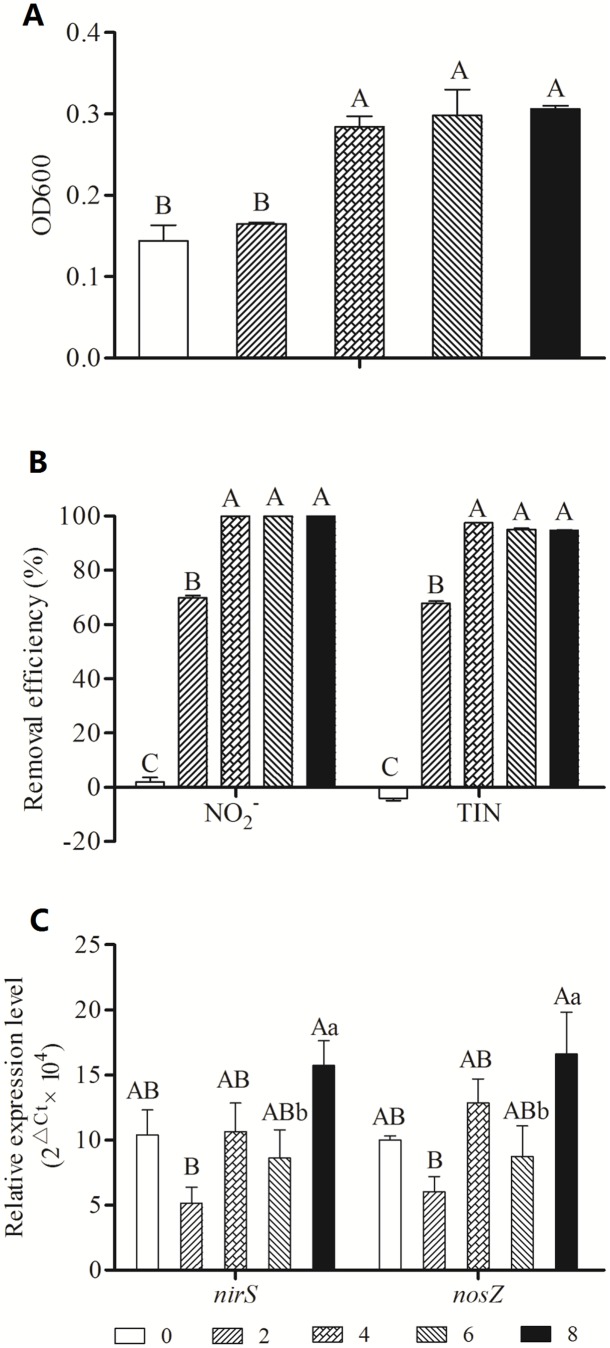
Effects of C/N on the growth (A), denitrification (B) and gene expression (C) of SC221-M.

When the C/N ratio was 4, 6 or 8, the OD_600_ and TIN were significantly higher than when the C/N ratio was 0 or 2. The corresponding nitrite and TIN removal rates were greater than 99.9% and 94% (Fig. 3A, 3B), respectively. The C/N ratios can be ranked according to the relative expression levels of *nirS* and *nosZ* as follows: 8>4>0>6>2 (Fig. 3C).

The organic carbon source plays an essential role in the growth and denitrification of *P. stutzeri* SC221-M. When the C/N is zero, the bacteria cannot use an external carbon source as an electron donor for denitrification. However, as the C/N ratio increases, the carbon source will no longer be a limiting factor for bacterial growth and denitrification.

The optimum C/N ratio range was determined to be 4–8 in the present study, which is consistent with data reported by Kim *et al.* (2008). For some strains, different carbon sources correspond to different optimum C/N ratios [25].

### 3.4 Effects of temperature on growth, denitrification and gene expression

**Figure 4:**
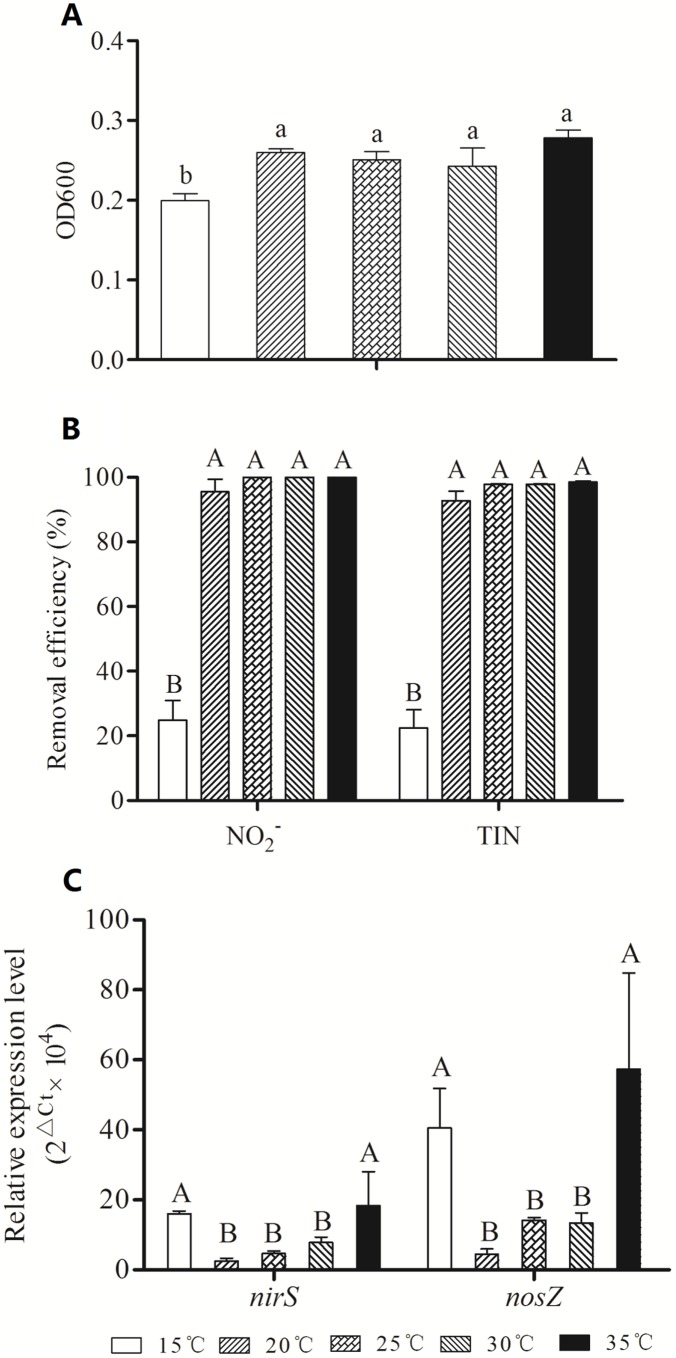
Effects of temperature on growth (A), denitrification (B) and gene expression (C) in SC221-M.

SC221-M did not grow well at 15°C (Fig. 4A). The nitrite and TIN removal rates were greater than 95.45% and 92.68% at 20, 25, 30 and 35°C, which were significantly higher than those at 15°C (Fig. 4B). However, the relative expression levels of *nirS* and *nosZ* at 15°C were significantly higher than those at 20, 25 and 30°C (Fig. 4C).

The growth and nitrogen removal rates of SC221-M were clearly inhibited at low temperatures, but relatively high temperatures had little effect. Low temperatures affect the enzyme activity of *P. stutzeri*, slowing its growth rate and lowering the nitrogen removal rate. However, the effect of temperature on the expression of the denitrification genes is quite different. *nirS* and *nosZ* expression at 15°C and 35°C were higher than under the other temperature conditions. Our results are not consistent with the research of Saleh-Lakha *et al*. (2009) [26], who found that incubation at 20°C promotes the expression of the *nirS* gene in *P. mandelii* to a greater extent than incubation at 10°C or 30°C. Further research is needed to understand why low temperatures promote the expression of denitrification genes.

### 3.5 Water quality control

**Figure 5:**
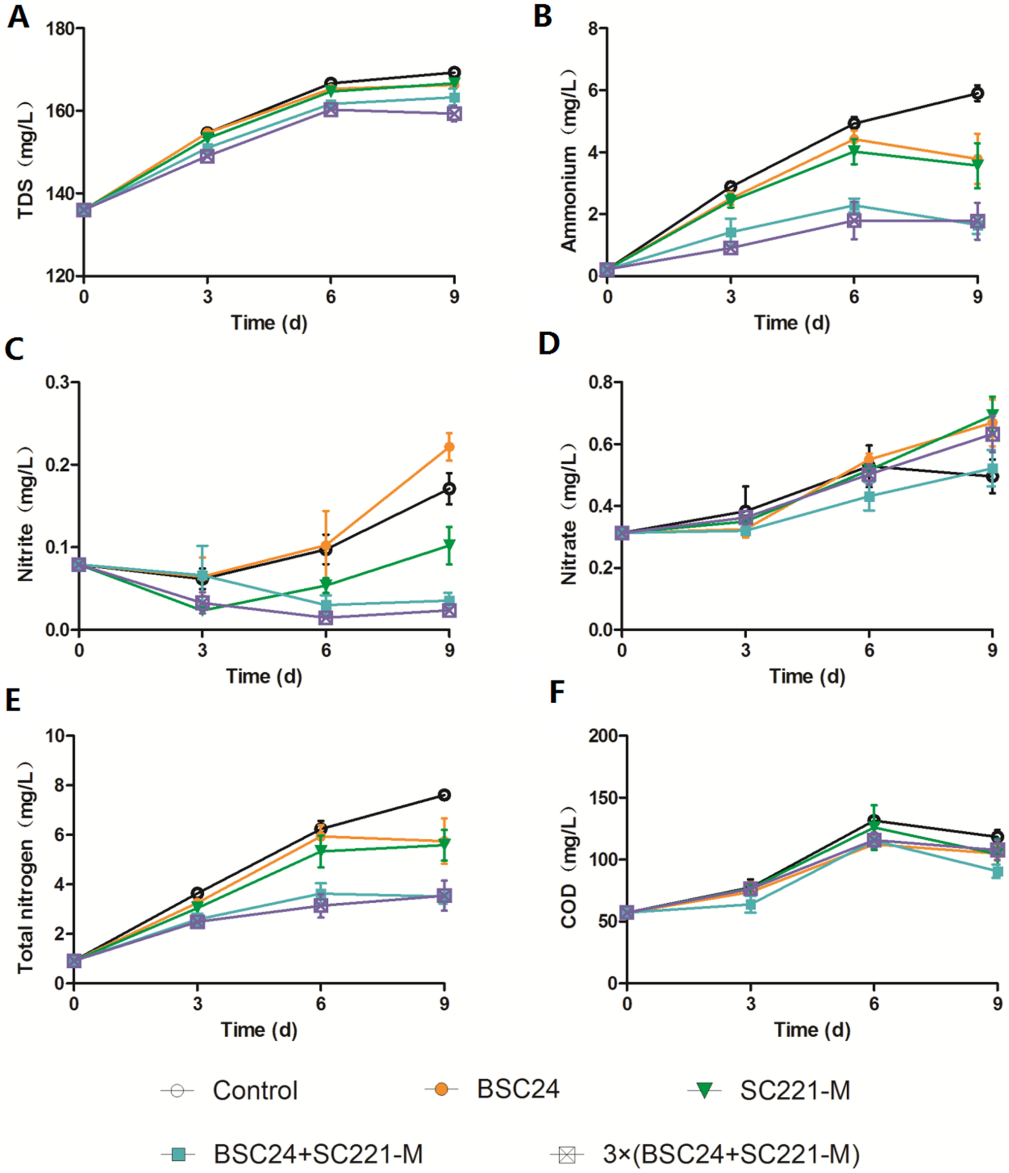
Effects of adding different microbial preparations on the concentrations of TDS (A), ammonium (B), nitrite (C), nitrate (D), total nitrogen (E) and COD (F) in the water of an experimental grass carp aquaculture.

The test groups can be ranked based on the concentrations of TDS, ammonium and total nitrogen in the aquaculture water from high to low as follows (Fig. 5): control> BSC24, and SC221-M> mixed preparation>3× mixed preparation (*P<*0.01).

The nitrogen removal rates were highest on day 9 after adding the preparations. The removal rates in the BSC24, SC221-M, mixed preparation and the 3× mixed preparation groups were 24.5%, 26.6%, 53.9% and 53.4%, respectively. On day 9, the nitrite concentration in the BSC24 group was 0.22±0.01 mg/L, which was 29.6% higher than in the control group (*P<*0.01). The BOD of the 3× mixed preparation group was 20.6% lower than that of the control group (*P<*0.01).

An excess of aqueous ammonia, nitrite and COD harms the health of aquatic animals [2], [3]. By adding microbial preparations to aquaculture water, the levels of ammonia, nitrite, total nitrogen and COD were decreased to some extent. These data are consistent with the results of previous studies [27], [28]. Different bacteria show different denitrification characteristics. Some strains directly reduce nitrate into nitrogen, without accumulating nitrite, while others produce a low concentration of nitrite during denitrification [22]. Our results showed that the addition of BSC24 led to the accumulation of nitrate and nitrite, while the other preparations did not.

There are advantages to using mixed preparations instead of using BSC24 or SC221-M alone. The mixed preparation and the 3× mixed preparation were not significantly different, nor were the BSC24 and SC221-M preparations. These results indicate that the interaction between SC221-M and BSC24 is important for improving water quality.

Why is a mixed preparation containing BSC24 and SC221-M more effective than either strain alone? There are many possible reasons. First, both *P. stutzeri* and *B. cereus* are capable of breaking down pollutants and promoting water restoration. *P. stutzeri* is a model strain for studying denitrification [29]. Various enzymes are involved in nitrification and denitrification in *P. stutzeri*, and this species can use a variety of organic compounds as carbon sources for denitrification. *B. cereus* is widely distributed throughout a variety of environments, is highly adaptable, and can form spores, allowing it to easily dominant the microbial environment. In addition, *B. cereus* BSC24 produces more nitrite, which both provides more nitrogen for denitrification and enhances the expression of genes encoding denitrification enzymes. By mixing BSC24 and SC221-M together, we can take full advantage of each strain and obtain better results. A similar effect is observed when using Effective Microorganisms, which is composed of a variety of microorganisms, including photosynthetic bacteria, lactic acid bacteria, actinomycetes, yeast, and fungi. These organisms complement each other and exhibit a wide range of agricultural applications [30].

### 3.6 Microbial community structure

**Figure 6:**
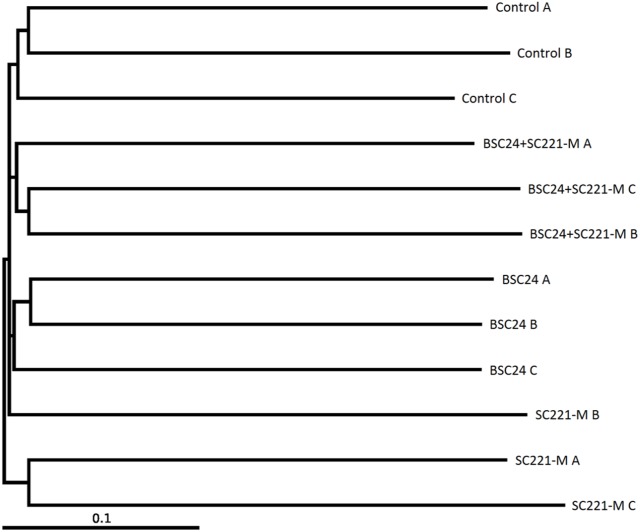
UPGMA analysis of samples from the control, BSC24, SC221-M, and mixed preparation (BSC24+SC221-M) groups.

**Figure 7:**
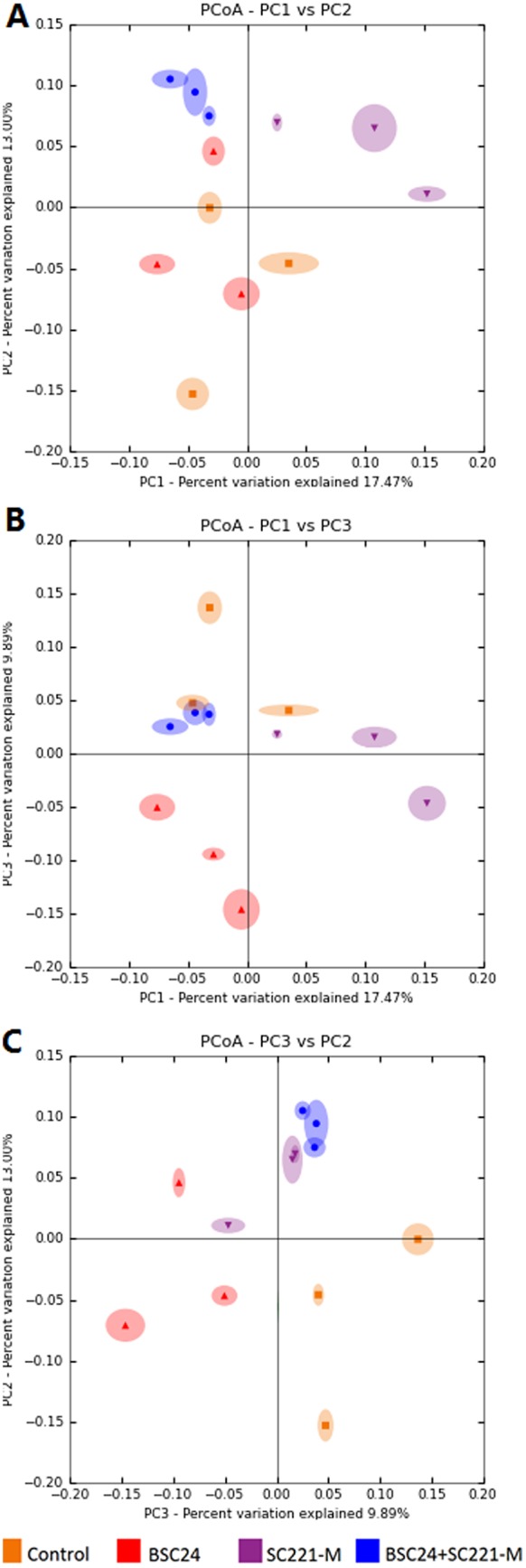
Principal coordinates analyses of samples from the control (orange), BSC24 (red), SC221-M (purple), and mixed preparation (BSC24+SC221-M, blue) groups.

UPGMA analysis indicated that all of the experimental groups, including the controls, formed separate clusters (Fig. 6). Through principal coordinates analysis, we found the smallest differences between samples in the mixed preparation group, followed by the SC221-M group. The correlations between the groups and the principle components are shown in Fig. 7.

**Figure 8:**
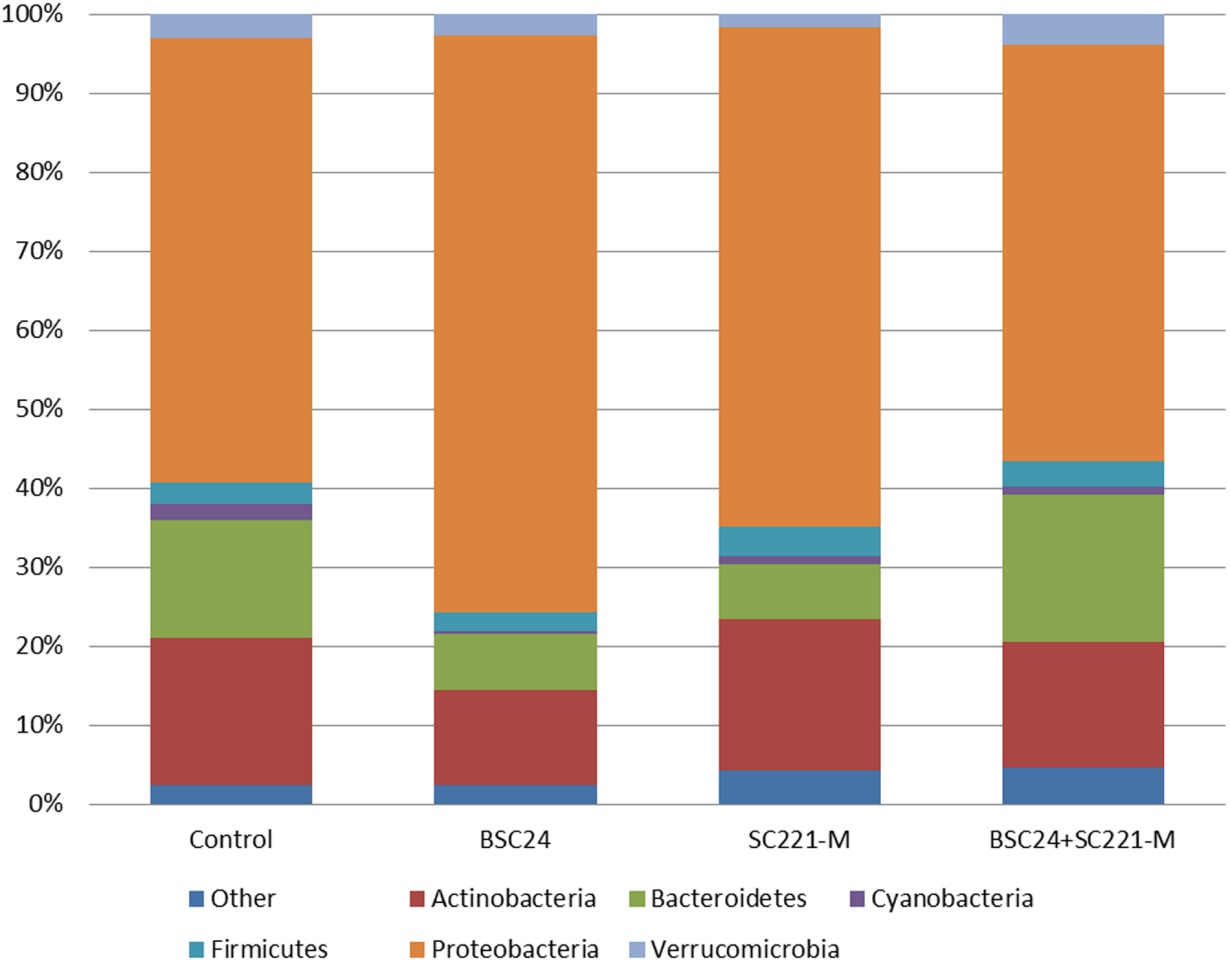
Comparison of microbial community structure at the phylum level.

All of the groups were dominated by Proteobacteria. Compared with the control group, the proportion of Proteobacteria increased, while those of Bacteroidetes and Actinobacteria decreased in the BSC24 group. The proportion of Proteobacteria also increased SC221-M group, while that of Bacteroides decreased. Finally, in the mixed preparation group, the proportion of Bacteroides and Verrucomicrobia increased while that of Actinobacteria decreased (Fig. 8).

**Figure 9:**
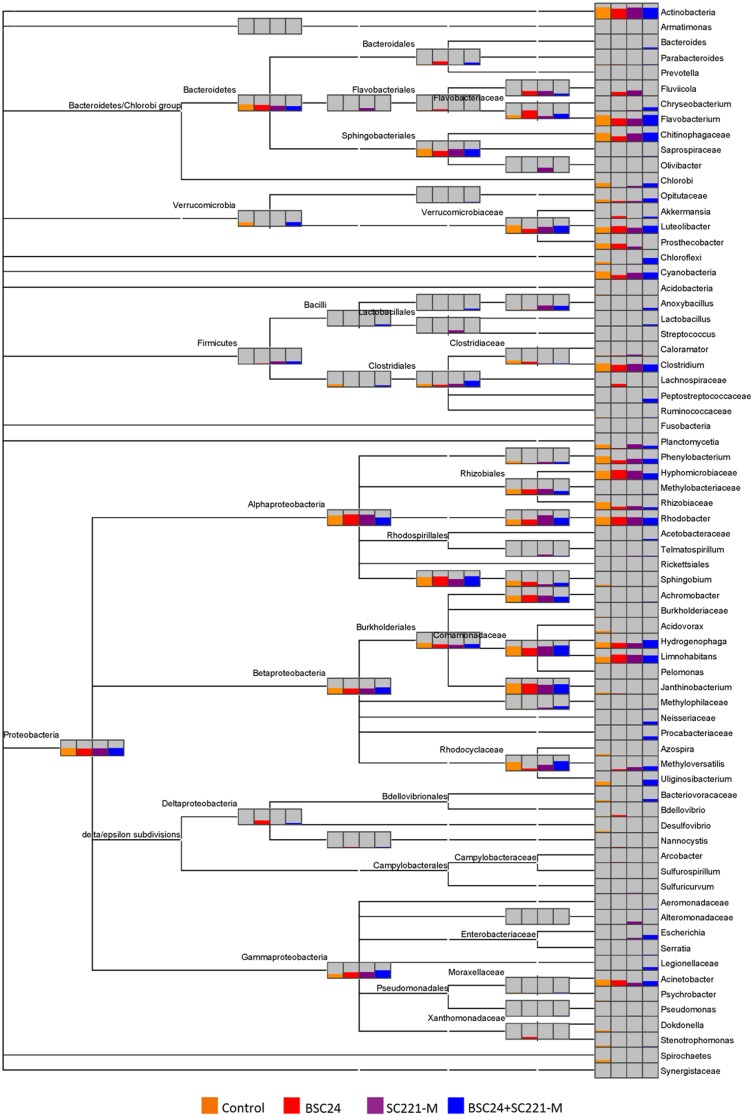
A detailed chart for the comparison of microbial structure.

Through a detailed comparison of the microbial communities, we found that the number of bacterial species present increased significantly after adding the mixed preparation. The group-specific bacterial species in the mixed preparation group included Saprospiraceae, Anoxybacillus, Lactobacillus, Peptostreptococcaceae, Acetobacteraceae, Methylophilaceae, Sulfurospirillum and Serratia. Significant changes in the microbial community structure in the water occurred after adding the microbial preparations (Fig. 9).

A large number of microbial species inhabit water and are essential for a healthy water environment. Many environmental factors can affect the diversity of microbial communities, including temperature, pH, and dissolved oxygen. In this study, the different treatment groups displayed different microbial communities.

Adding microbial preparations to aquaculture water significantly altered the structure of the microbial community, especially when using the mixed preparation. The number of bacterial species increased significantly after adding the mixed preparation. Our results demonstrate a synergy between BSC24 and SC221-M that is important for water quality control, and there was a close relationship between the microbial community structure and the hydrochemical parameters. Previous research has indicated that more than 150 different bacteria from more than 50 genera are capable of carrying out denitrification [31]. As microbial diversity was found to increase after adding the mixed preparation, the increased diversity may be associated with enhanced water denitrification.

These results are consistent with those of Zhang *et al*. (2013), who found that adding a *B. subtilis* preparation to grass carp water significantly increases the microbial diversity of the water [7], which may be the result of an improved aquaculture environment.

### 3.7 Genome of *P. stutzeri* SC221-M

The results of the GC-depth and k-mer distribution analyses revealed that the 15-mer distribution followed a Poisson distribution (S2 Figure). The scatter plot shown in S3 Figure was also similar to a Poisson distribution, and there was a peak corresponding to the GC content of the genome, indicating that the genome sequencing was not biased.

Based on the sequencing, assembly and analysis results, we obtained a correct and complete genome sequence for SC221-M. The NCBI database contains the complete genome sequences of 6 strains of *P. stutzeri*: A1501, DSM4166 [32], CGMCC1.1803, CCUG29243, RCH2 and DSM10701. Schematic diagrams of the genome are available for many *P. stutzeri* strains. The strains show differences in their genome size and structure that allow them to adapt to different environments. As shown in S4 Figure, SC221-M, A1501, DSM4166 and ATCC17588 are closely related, whereas SC221-M and CCUG29243 are more distantly related, and SC221-M and RCH2 are the most distantly related. These results indicate that SC221-M, A1501, and DSM4166 share a similar denitrification mechanism.

A1501 and DSM4166 are model nitrogen-fixing bacteria. As the nitrogen fixation mechanism is well understood in these strains, the relationship between nitrogen fixation and denitrification in SC221-M would be worth studying.

## Conclusions

Environmental factors can affect the growth and denitrification of *P. stutzeri* SC221-M. This strain shows high genomic sequence homology to the nitrogen-fixing *P. stutzeri* A1501 and DSM4166 strains, and these strains share a similar denitrification mechanism. The addition of microbial preparations to an experimental aquaculture system can reduce pollutants, leading to improvements in both water quality and the structure of the microbial community, as there is a close relationship between microbial community structure and hydrochemical parameters. These results indicate that *P. stutzeri* SC221-M and its related microbial preparations are potential candidates for use in the regulation of water quality in commercial aquaculture systems. Future studies will examine the mobilization of the preparations and the potential applications for circulating aquaculture systems or aquaculture farms.

## Supporting Information

S1 Figure **Morphological features of** ***P. stutzeri*** **SC221-M (A) and** ***B. cereus*** **BSC24 (B).**
(TIF)

S2 Figure **15-mer analysis of the genomic sequence of SC221-M.** The X-coordinate is depth, and the Y-coordinate is proportion. Regardless of the sequencing error, genome heterozygosity and duplication, the 15-mer distribution should follow a Poisson distribution. However, low-depth k-mers account for a large proportion due to sequencing errors.
(TIF)

S3 Figure **GC content and depth correlation analysis for SC221-M.** The X-coordinate is the GC content, and the Y-coordinate is the average depth. By calculating the GC content and the average depth using a window of 500 bp, we can analyze whether GC bias exists. In the absence of GC bias, the scatter diagram should resemble a Poisson distribution. The higher the deviation from the peak near the GC content of the genome, the lower the depth.
(TIF)

S4 Figure **Phylogenic tree of** ***P. stutzeri*** **SC221-M and other strains.**
(TIF)

S1 Table **Experimental design.**
(DOCX)

S2 Table **Primers for qPCR.**
(DOCX)

S3 Table **Ingredients and nutritional composition of the basal diet.** The premix provides the following per kilogram of food: Vitamin A, 150,000 IU; vitamin D3, 30,000 IU; vitamin E, 750 mg; vitamin K3, 150 mg; Fe 2.5 g, Cu 0.075 g, Zn 0.75 g, Mn 0.5 g, Mg 5 g, I 22.5 mg, Se 3.5 mg, Co 7.5 mg. The feed doesn’t contain any antibiotics or probiotics.
(DOCX)

S4 Table **Primers for the V3 region of the 16S rRNA gene.**
(DOCX)

S5 Table **Assembling statistics for the SC221-M genome sequence.** The second column shows the number of scaffolds longer than 500 bp, and the third column shows the number of contigs obtained by breaking the scaffolds from the second column.
(DOCX)

S6 Table **SNP annotation statistics compared with the reference strain DSM4166.** Start_syn, start codon synonymous mutation; Stop_syn, stop codon synonymous mutation; Start_nonsyn, start codon non-synonymous mutation; Stop_nonsyn, stop codon non-synonymous mutation; Premature_stop, a triplet codon mutated into a stop codon; Synonymous, synonymous mutation in the coding region; Nonsynonymous, non-synonymous mutation in the coding region; Intergenic, SNPs in the noncoding region.
(DOCX)

S1 File **Genome sequencing, assembly, SNP detection and phylogenetic tree construction.**
(DOCX)
