# Supplementary figures and images for: The Denitrification Characteristics of Pseudomonas stutzeri SC221-M and Its Application to Water Quality Control in Grass Carp Aquaculture
Source: PLoS One. 2014 Dec 9;9(12):e114886. doi: 10.1371/journal.pone.0114886 (PMC4260960; doi:10.1371/journal.pone.0114886)

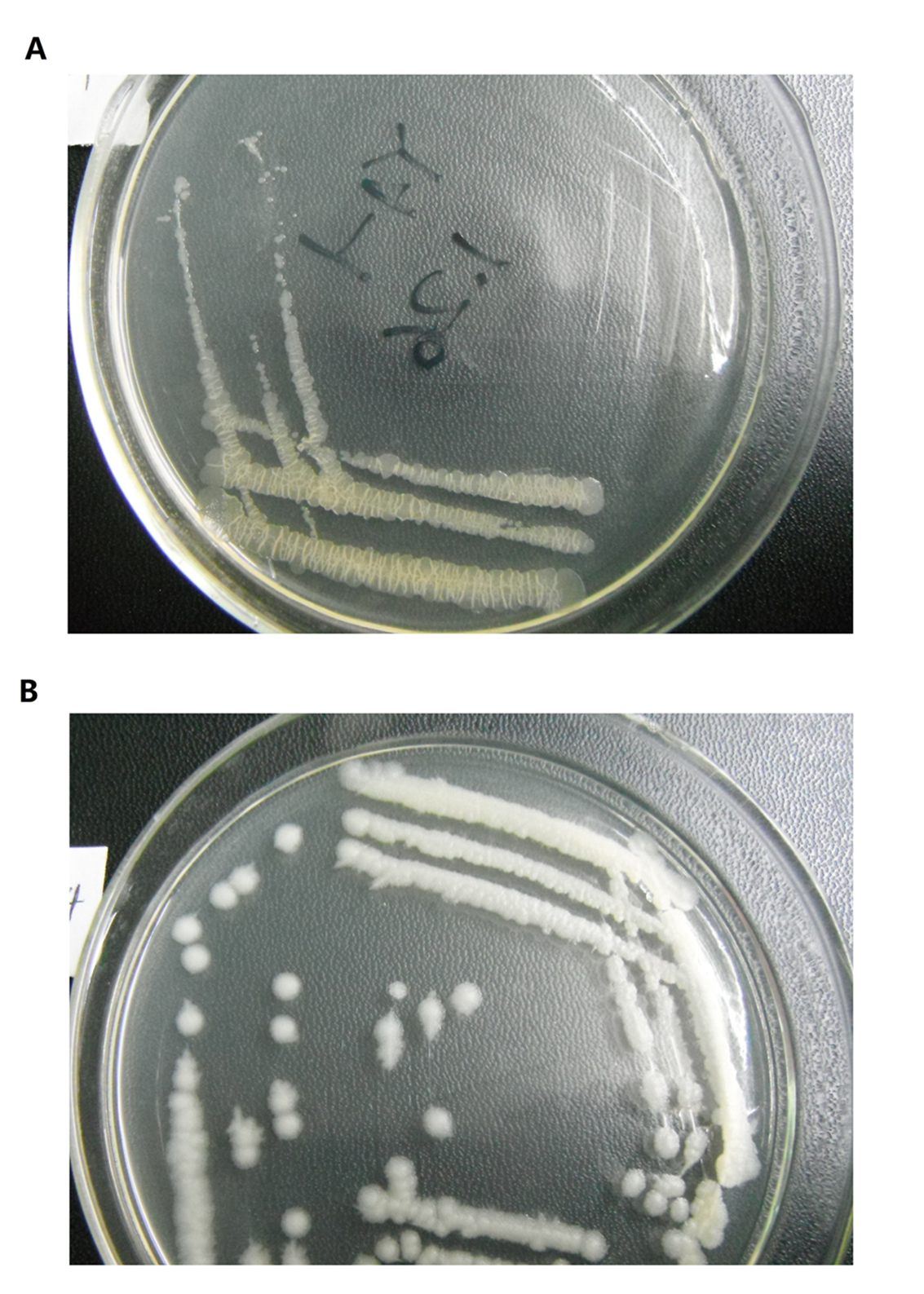

Supplement: S1 Figure — Morphological features of P. stutzeri SC221-M (A) and B. cereus BSC24 (B). (TIF) [file pone.0114886.s001.tif]

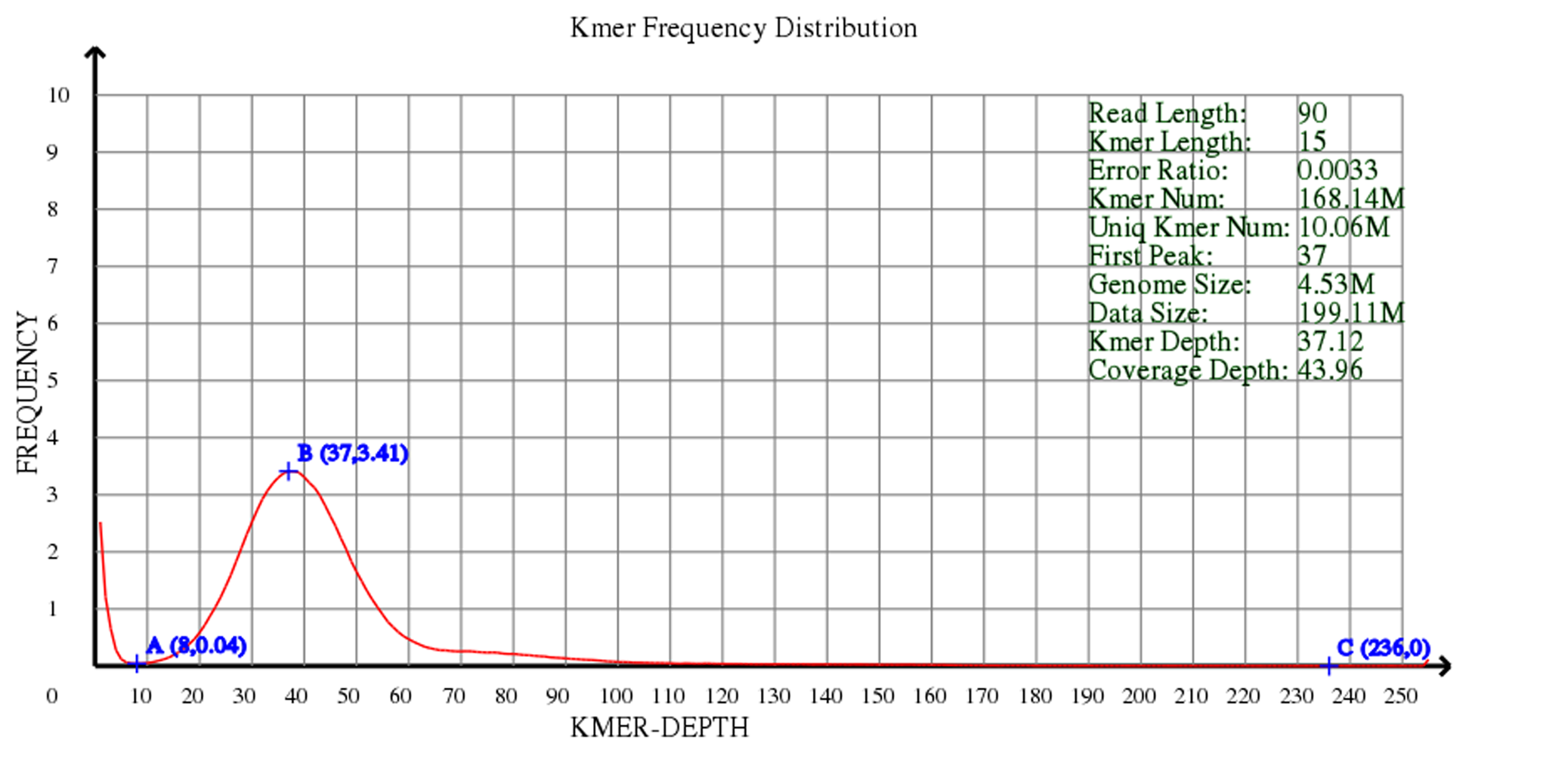

Supplement: S2 Figure — 15-mer analysis of the genomic sequence of SC221-M. The X-coordinate is depth, and the Y-coordinate is proportion. Regardless of the sequencing error, genome heterozygosity and duplication, the 15-mer distribution should follow a Poisson distribution. However, low-depth k-mers account for a large proportion due to sequencing errors. (TIF) [file pone.0114886.s002.tif]

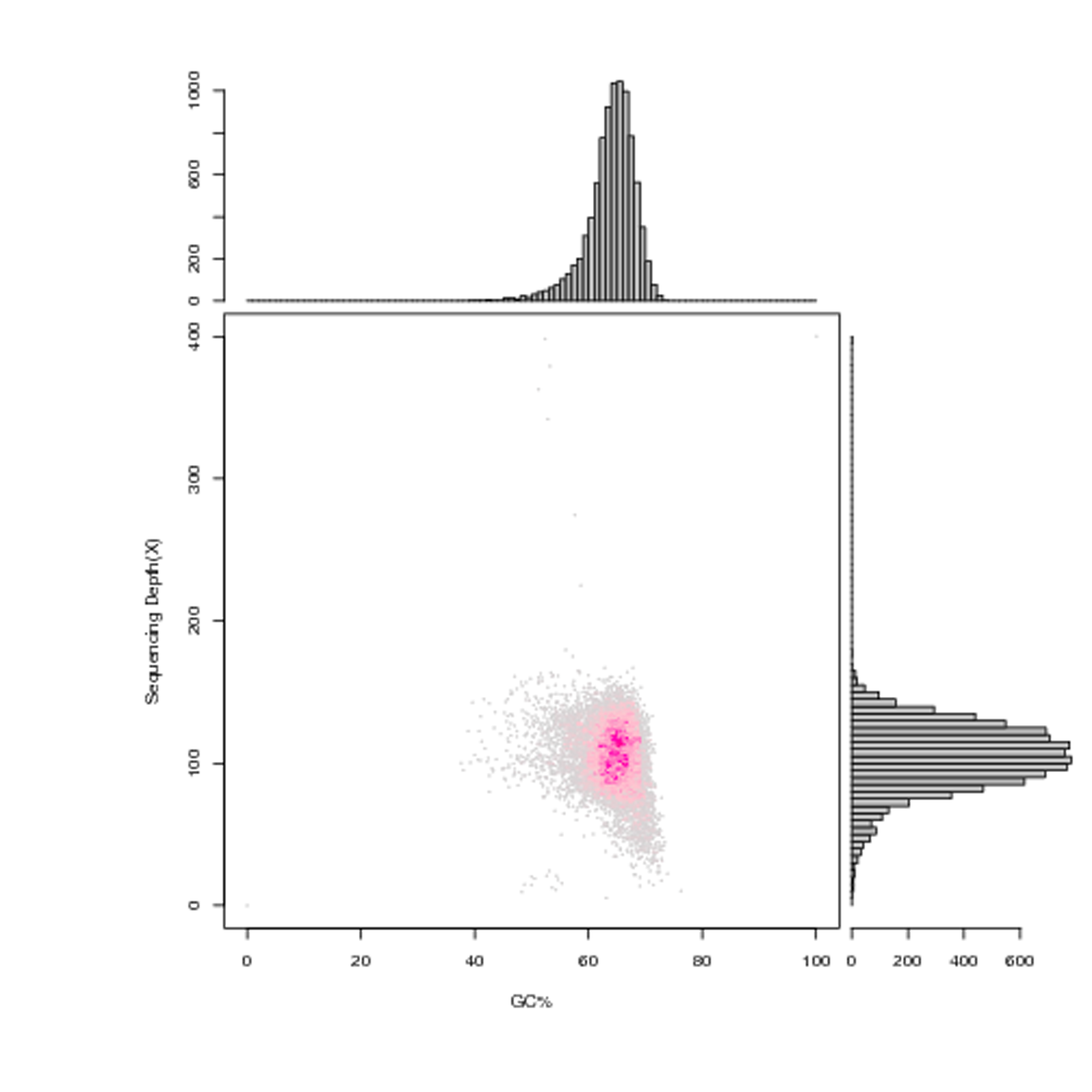

Supplement: S3 Figure — GC content and depth correlation analysis for SC221-M. The X-coordinate is the GC content, and the Y-coordinate is the average depth. By calculating the GC content and the average depth using a window of 500 bp, we can analyze whether GC bias exists. In the absence of GC bias, the scatter diagram should resemble a Poisson distribution. The higher the deviation from the peak near the GC content of the genome, the lower the depth. (TIF) [file pone.0114886.s003.tif]

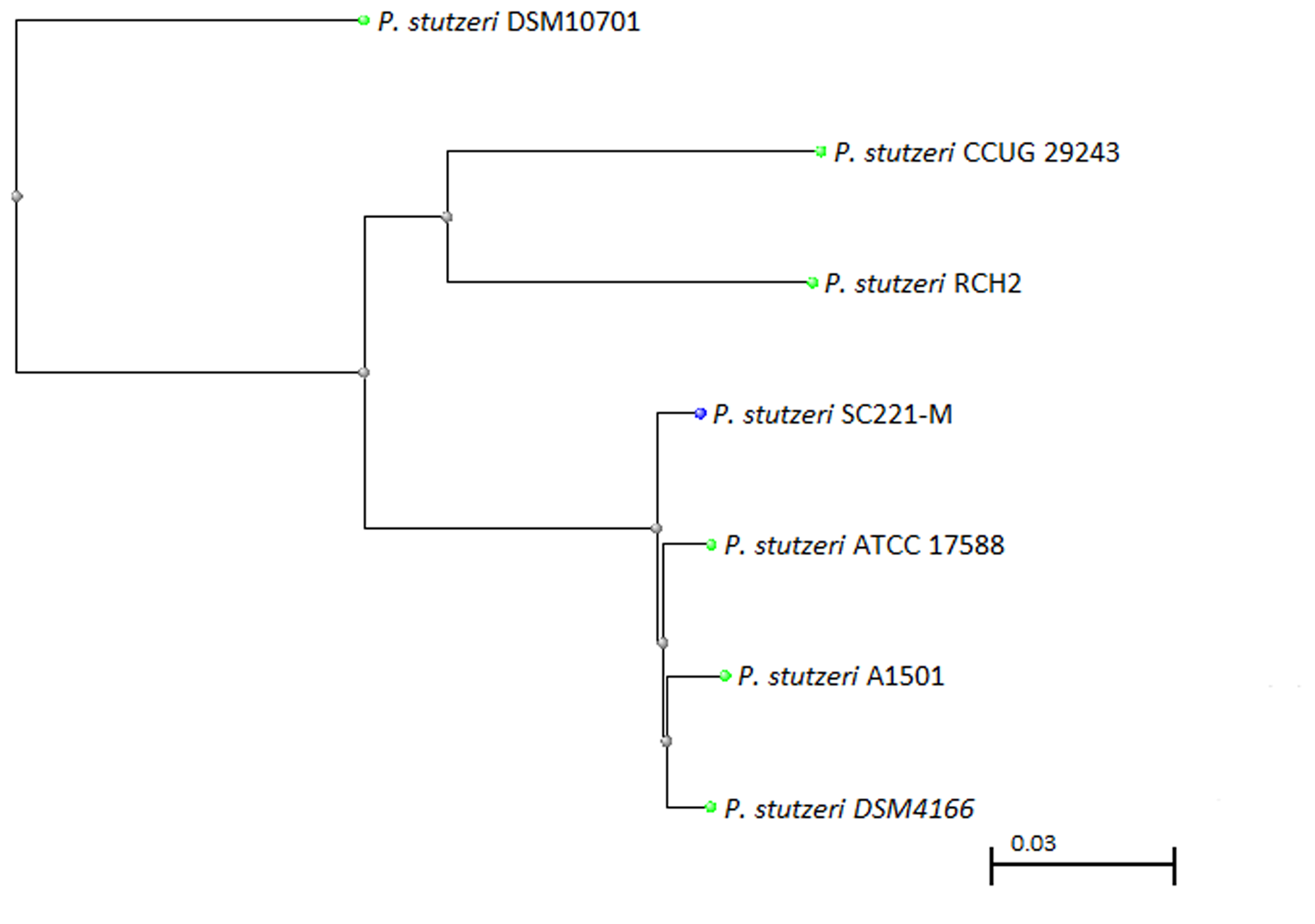

Supplement: S4 Figure — Phylogenic tree of P. stutzeri SC221-M and other strains. (TIF) [file pone.0114886.s004.tif]
