# Supplementary material for: The Denitrification Characteristics of Pseudomonas stutzeri SC221-M and Its Application to Water Quality Control in Grass Carp Aquaculture
Source: PLoS One. 2014 Dec 9;9(12):e114886. doi: 10.1371/journal.pone.0114886 (PMC4260960; doi:10.1371/journal.pone.0114886)
Supplement: S1 Table — Experimental design. (DOCX) [file pone.0114886.s005.docx]

**Table S1. Experimental design.**

| **Trail** | **Environmental factor** | **Design** |
| --- | --- | --- |
| 1 | Nitrogen sources | Sodium citrate was used as the carbon source, and NaNO_2_, NaNO_3_, or NH_4_Cl was used as a nitrogen source (nitrogen concentration, 100 mg/L); C/N, = 10; incubated at 30 °C for 24 h. |
| 2 | Carbon sources | NaNO_2_ was used as the nitrogen source (nitrogen concentration, 100 mg/L), and sodium citrate, glucose, or starch was used as a carbon source; C/N = 10; incubated at 30 °C for 24 h. |
| 3 | C/N | NaNO_2_ was used as the nitrogen source (nitrogen concentration, 100 mg/L), and sodium citrate was used as the carbon source; C/N = 0, 2, 4, 6 or 8; incubated at 30 °C for 24 h. |
| 4 | Temperature | NaNO_2_ was used as the nitrogen source (nitrogen concentration, 100 mg/L), and sodium citrate was used as the carbon source; C/N = 8; incubated at 15, 20, 25 or 30 °C for 24 h. |
