# Supplementary material for: The Denitrification Characteristics of Pseudomonas stutzeri SC221-M and Its Application to Water Quality Control in Grass Carp Aquaculture
Source: PLoS One. 2014 Dec 9;9(12):e114886. doi: 10.1371/journal.pone.0114886 (PMC4260960; doi:10.1371/journal.pone.0114886)
Supplement: S2 Table — Primers for qPCR. (DOCX) [file pone.0114886.s006.docx]

**Table S2. Primers for qPCR.**

| Gene | Accession no. | Oligo（5'→3'） | Length  (bp) | Tm  (°C) | |
| --- | --- | --- | --- | --- | --- |
| *16S* | JX008025 | GGGCTCAACCTGGGAACTGC | 124 | | 63 |
|  |  | GTGGTCGCCTTCGCCACTG |  |  |  |
| *nirS* | X16452 | GGCAACCTGTTCGTCAAGAC | 137 | | 63 |
|  |  | CGGAGTCCTTGGCGACGT |  |  |  |
| *nosZ* | HE814032 | GCGCTGTCCAAGTTCTCCAAGG | 82 | | 63 |
|  |  | CGGAGATGTCGATCAACTGGTCGTT |  |  |  |
