# Supplementary material for: The Denitrification Characteristics of Pseudomonas stutzeri SC221-M and Its Application to Water Quality Control in Grass Carp Aquaculture
Source: PLoS One. 2014 Dec 9;9(12):e114886. doi: 10.1371/journal.pone.0114886 (PMC4260960; doi:10.1371/journal.pone.0114886)
Supplement: S3 Table — Ingredients and nutritional composition of the basal diet. The premix provides the following per kilogram of food: Vitamin A, 150,000 IU; vitamin D3, 30,000 IU; vitamin E, 750 mg; vitamin K3, 150 mg; Fe 2.5 g, Cu 0.075 g, Zn 0.75 g, Mn 0.5 g, Mg 5 g, I 22.5 mg, Se 3.5 mg, Co 7.5 mg. The feed doesn’t contain any antibiotics or probiotics. (DOCX) [file pone.0114886.s007.docx]

**Table S3. Ingredients and nutritional composition of the basal diet.**

| Ingredients | Composition (%) | Nutritional index | Proximate composition  (% wet weight) |
| --- | --- | --- | --- |
| Fish meal | 1.0 | Moisture | 9.31 |
| Soybean meal | 5.0 | Crude protein | 28.40 |
| Cottonseed meal | 23.8 | Crude fat | 2.97 |
| Rapeseed meal | 27.2 | Crude ash | 12.08 |
| Wheat flour | 18.0 | Calcium | 0.80 |
| Rice bran | 8.0 | Total phosphonium | 1.29 |
| Sake lees powder | 6.0 |  |  |
| Malt root | 6.0 |  |  |
| Premix | 5.0 |  |  |
