# Supplementary material for: The Denitrification Characteristics of Pseudomonas stutzeri SC221-M and Its Application to Water Quality Control in Grass Carp Aquaculture
Source: PLoS One. 2014 Dec 9;9(12):e114886. doi: 10.1371/journal.pone.0114886 (PMC4260960; doi:10.1371/journal.pone.0114886)
Supplement: S4 Table — Primers for the V3 region of the 16S rRNA gene. (DOCX) [file pone.0114886.s008.docx]

**Table S4. Primers for the V3 region of the 16S rRNA gene.**

| Primer | Oligo（5'→3'） |
| --- | --- |
| Control A-Forward | ACGAGTGGACACGGTCCAGACTCCTACGG |
| Control B-Forward | ACGCTCGGACACGGTCCAGACTCCTACGG |
| Control C-Forward | AGACGCAGACACGGTCCAGACTCCTACGG |
| BSC24 A-Forward | AGCACTGGACACGGTCCAGACTCCTACGG |
| BSC24 B-Forward | ATCAGACGACACGGTCCAGACTCCTACGG |
| BSC24 C-Forward | ATATCGCGACACGGTCCAGACTCCTACGG |
| SC221-M A-Forward | TCTCTATGACACGGTCCAGACTCCTACGG |
| SC221-M B-Forward | TGATACGGACACGGTCCAGACTCCTACGG |
| SC221-M C-Forward | TACTGAGGACACGGTCCAGACTCCTACGG |
| BSC24 + SC221-M A- Forward | TCACGTGGACACGGTCCAGACTCCTACGG |
| BSC24 + SC221-M B- Forward | TGCATCTGACACGGTCCAGACTCCTACGG |
| BSC24 + SC221-M C- Forward | TAGCACAGACACGGTCCAGACTCCTACGG |
| Reverse primer | GTATTACCGCGGCTGCTGGCAC |
