# Supplementary material for: The Denitrification Characteristics of Pseudomonas stutzeri SC221-M and Its Application to Water Quality Control in Grass Carp Aquaculture
Source: PLoS One. 2014 Dec 9;9(12):e114886. doi: 10.1371/journal.pone.0114886 (PMC4260960; doi:10.1371/journal.pone.0114886)
Supplement: S5 Table — Assembling statistics for the SC221-M genome sequence. The second column shows the number of scaffolds longer than 500 bp, and the third column shows the number of contigs obtained by breaking the scaffolds from the second column. (DOCX) [file pone.0114886.s009.docx]

**Table S5. Assembling statistics for the SC221-M genome sequence.**

|  | **Scaffold** | **Contig** |
| --- | --- | --- |
| **Total Number** | 131 | 399 |
| **Total Length (bp)** | 4,545,027 | 4,526,713 |
| **N50 (bp)** | 87,009 | 26,899 |
| **N90 (bp)** | 19,680 | 7,163 |
| **Max Length (bp)** | 224,506 | 180,380 |
| **Min Length (bp)** | 542 | 201 |
| **GC Content (%)** | 64.01 | 64.01 |
