# Supplementary material for: The Denitrification Characteristics of Pseudomonas stutzeri SC221-M and Its Application to Water Quality Control in Grass Carp Aquaculture
Source: PLoS One. 2014 Dec 9;9(12):e114886. doi: 10.1371/journal.pone.0114886 (PMC4260960; doi:10.1371/journal.pone.0114886)
Supplement: S6 Table — SNP annotation statistics compared with the reference strain DSM4166. Start_syn, start codon synonymous mutation; Stop_syn, stop codon synonymous mutation; Start_nonsyn, start codon non-synonymous mutation; Stop_nonsyn, stop codon non-synonymous mutation; Premature_stop, a triplet codon mutated into a stop codon; Synonymous, synonymous mutation in the coding region; Nonsynonymous, non-synonymous mutation in the coding region; Intergenic, SNPs in the noncoding region. (DOCX) [file pone.0114886.s010.docx]

**Table S6. SNP annotation statistics compared with the reference strain DSM4166.**

| **Region** | **Type** | **Number** | **Percentage (%)** |
| --- | --- | --- | --- |
| **CDS** | Start_syn | 2 | 0.0033 |
|  | Stop_syn | 29 | 0.0478 |
|  | Start_nonsyn | 17 | 0.028 |
|  | Stop_nonsyn | 15 | 0.0247 |
|  | Premature_stop | 50 | 0.0825 |
|  | Synonymous | 43,396 | 71.5621 |
|  | Nonsynonymous | 10,743 | 17.7157 |
|  | Total | 54,213 | 89.3999 |
| **Intergenic** | | 6,428 | 10.6001 |
| **Total** | | 60,641 | -- |
