# Supplementary material for: The Denitrification Characteristics of Pseudomonas stutzeri SC221-M and Its Application to Water Quality Control in Grass Carp Aquaculture
Source: PLoS One. 2014 Dec 9;9(12):e114886. doi: 10.1371/journal.pone.0114886 (PMC4260960; doi:10.1371/journal.pone.0114886)
Supplement: S1 File — Genome sequencing, assembly, SNP detection and phylogenetic tree construction. (DOCX) [file pone.0114886.s011.docx]

**Supporting Information**

**S1 Genome sequencing, assembly, SNP detection and phylogenetic tree construction**

**S1.1 Genome sequencing and analysis**

LB broth was inoculated with a single colony of SC221-M and incubated at 37 °C and 200 rpm for 18 h. Genomic DNA was then extracted and stored in TE buffer. Subsequently, the genomic DNA was sent to BGI-Shenzhen, China, for genome sequencing.

The short reads were assembled into genomic sequences using SOAPdenovo (version 2.04). The assembly result was locally assembled and optimized according to paired-end and overlap relationships by mapping reads to Contig and Scaffold, after which the GC-depth and k-mer distribution (k = 15) were analyzed ([Li *et al*., 2008](#_ENREF_4); [Li *et al*., 2010](#_ENREF_5)).

SNPs were detected based on the alignment ([Yu *et al*., 2011](#_ENREF_7)) between the assembly result and the reference genome (*P. stutzeri* DSM4166).

Each query sequence was aligned with the reference sequence using the alignment software MUMmer (version 3.22). The variable sites between the query sequence and the reference sequence were identified and filtered prior to detecting potential SNP sites ([Kurtz *et al.*, 2004](#_ENREF_3)).

Sequences of 100 bp on both sides of SNPs in the reference sequence were extracted and aligned with the assembly results to verify the SNP sites using BLAT ([de Leeuw *et al*., 2012](#_ENREF_2)). If the length of the aligned sequences was shorter than 101 bp, the SNP was counted as an outlier and removed; if the extracted sequence could be aligned with the assembly results several times, the SNP was considered to be located in the repeat region and was also removed.

Blast (version 2.2.2), TRF (version 4.04), and Repeatmask (version 3.2.9) were used to predict SNPs in repeat regions ([Benson, 1999](#_ENREF_1); [Schmid, 1996](#_ENREF_6)). The credible SNPs were obtained by filtering SNPs located in repeat regions.

The genome of SC221-M was compared with other strains using genome BLAST at NCBI (National Center for Biotechnology Information). A1501, DSM4166, ATCC17588, CCUG29243, RCH2 and DSM10701 were selected as reference strains.

**S1.2 Results**

Based on the assembly results, the size of the SC221-M genome was found to be 4,545,027 bp, with a 64.01% GC content; 131 scaffolds containing 399 contigs were identified (Table S5). The genome of SC221-M harbored 60,641 SNPs, including 43,396 synonymous mutations and 10,743 non-synonymous mutations, compared with the reference strain DSM4166 (Table S6).

**References**

Benson G (1999) Tandem repeats finder: a program to analyze DNA sequences. Nucleic Acids Res 27: 573-580.

de Leeuw N, Dijkhuizen T, Hehir-Kwa JY, Carter NP, Feuk L, et al. (2012) Diagnostic interpretation of array data using public databases and internet sources. Human mutation 33: 930-940.

Kurtz S, Phillippy A, Delcher AL, Smoot M, Shumway M, et al. (2004) Versatile and open software for comparing large genomes. Genome Biol 5: R12.

Li R, Li Y, Kristiansen K, Wang J (2008) SOAP: short oligonucleotide alignment program. Bioinformatics 24: 713-714.

Li R, Zhu H, Ruan J, Qian W, Fang X, et al. (2010) De novo assembly of human genomes with massively parallel short read sequencing. Genome Res 20: 265-272.

Schmid CW (1996) Alu: structure, origin, evolution, significance and function of one-tenth of human DNA. Prog Nucleic Acid Res Mol Biol 53: 283-319.
